# Cologne Consensus Conference 2016: assessment in accredited CME/CPD, 16 and 17 September 2016, Cologne, Germany

**DOI:** 10.1080/21614083.2017.1302671

**Published:** 2017-03-26

**Authors:** Ron Murray

**Affiliations:** ^a^ Independent CME/CPD Consultant, Pickering, UK

**Keywords:** CME/CPD, assessment, evaluation, simulation, outcomes, accreditation, provider

## Abstract

Participants and faculty members from around Europe and North America met in Cologne Germany on 16 and 17 September 2016, for the fifth annual Cologne Consensus Conference under the auspices of the European Cardiology Section Foundation (ECSF) in cooperation with various European and North American professional medical associations, accrediting bodies and CME providers. The conference was preceded by an afternoon workshop, organised by the European Board for Accreditation in Cardiology (EBAC) that allowed participants to observe a range of current e-learning modules in CME and to discuss implications for the accreditation of micro-e-learning. The conference theme was Assessment and the first day’s presentations covered Assessment Methods, Needs Assessment, Assessment of Interprofessional Teams, and Assessment of Providers. The second day’s topics were Assessment of Knowledge, Assessment of Practical Skills, Evaluation methods and the link between Assessment and Licensure. A diverse range of professional expertise among participants from both sides of the Atlantic provided stimulating discussion to make the conference a rewarding experience for all concerned.

The Cologne Consensus Conference 2016, under the auspices of the European Cardiology Section Foundation (ECSF) in cooperation with a number of European and North American professional medical associations, accrediting bodies and CME providers (see ECSF website http://e-cs-f.org/files/ecsf_16_ccc_programm_final.pdf) was held on 16 and 17 September 2016 In Cologne, Germany. The topic of “Assessments in Accredited CME/CPD” attracted participants and faculty members from around Europe and North America.

In a departure from previous years the main two-day conference was preceded by an afternoon workshop, organised by the European Board for Accreditation in Cardiology (EBAC) and chaired by Professor Reinhard Griebenow, which provided participants with the opportunity to observe a range of current e-learning modules in CME and to discuss implications for the accreditation of micro-e-learning. Representatives from organisations in Austria, USA and the Netherlands described e-learning materials in the specific areas of:

- ultrasound and echocardiography (https://123sonography.com/),
- cardiology (http://www.medconinternational.com/home),
- multilingual, multiplatform offerings with linked learning assessments (http://www.medscape.org/), and
- point of care micro e-learning (http://www.uptodate.com/home/uptodate-subscription-options-clinicians).

Discussion on the issues arising from the increasing availability of micro e-learning content focused on a list of topics such as:

- the logistics of awarding credit for materials of shorter lengths of time than the traditional one hour as the basis of a CME credit unit,
- legislative issues in different countries when trying to control how credits are awarded,
- control of content quality and relevance, and
- how to engage learners to participate in e-learning.

A consensus view emerged that a robust accreditation system such as that provided by EBAC could indeed provide the means for accreditation of micro e-learning modules and apply the same rigour as the criteria currently in place for live meetings.

## Day 1

Professor Heinz Weber, Chairman of ECSF Council introduced the conference by reviewing the topics addressed in the previous consensus conferences and highlighted recent links forged between accrediting bodies, particularly the agreement on mutual recognition of substantial equivalency between the US-based Accreditation Council for Continuing Medical Education (ACCME) and EBAC. In recognition of this recent agreement, a commemorative diploma was presented to Kate Regnier, Executive Vice-President of ACCME who was attending the conference as a faculty member. These collaborations were further exemplified by the news that the 2017 Consensus Conference will be presented in conjunction with the ACCME and the Royal College of Physicians and Surgeons of Canada which was also represented at the 2016 conference.

The main sessions of the consensus conference began with an introduction by Professor Reinhard Griebenow based on the thesis that assessments are the backbone of evidence-based accreditation and followed by a description of various scenarios of assessment in the field of CME/CPD. These scenarios included:

- activity organisers and faculty assessing participants and vice versa,
- accreditors assessing CPD providers, and
- professional organisations and licensing authorities assessing physicians.

This thesis was expanded by considering the target audience to be assessed as determining the data source(s) for assessment at individual and healthcare system levels. A range of pre-event learning assessment (needs assessment) methods was described such as periodic internal audits using electronic health records for comparison with guidelines or benchmarks. Discussion also focused on post-event evaluation and the need to find an acceptable compromise between affordability and practicability to provide meaningful information.

The main headings for the topics covered during Day 1 of the conference were as follows:

- assessment methods,
- research results,
- assessment of interprofessional teams, and
- assessment of providers.

## Assessment methods

This session was presented by Professor Jürgen Neuser who allowed participants to delve into the mechanics of testing as an assessment tool. Professor Neuser laid out various goals of testing in terms of:

- measuring performance,
- motivation and self-evaluation,
- evaluation of teaching, and
- being an instrument for sanctions.

**Figure 1.**
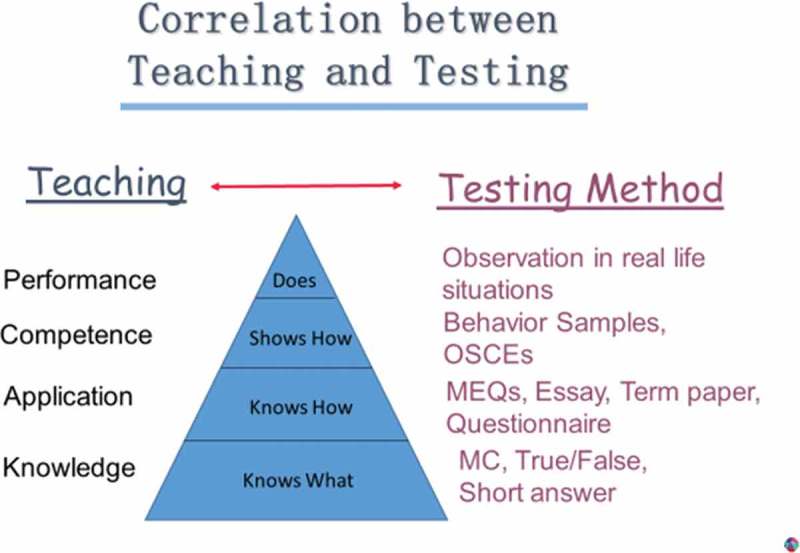
Teaching and testing correlation (adapted with permission from Professor Jürgen Neuser).

He outlined the taxonomy of learning domains and appropriate testing methods for various levels in Miller’s Pyramid of Learning (see Figure 1). Details of test planning, grading and evaluation completed this presentation.

Factors to consider in test planning were the determination of:

- test level,
- scope of test,
- weight of content, and
- weight levels.

Problems associated with grading were identified as:

- scaling,
- anchoring,
- standard setting, and
- complexity.

Evaluation dwelt on:

- difficulty,
- selectivity, and
- efficiency of alternatives.

in the pursuit of objectivity, reliability and validity.

Finally, testing was discussed in terms of test demands, evaluation and social behaviour so that all candidates can demonstrate their performance level. Discussion elicited comments on points such as not turning educators into testers and the benefits in CME of combining evaluation with real practice data whenever possible.

## Research studies

### Interdisciplinary knowledge

**Figure 2.**
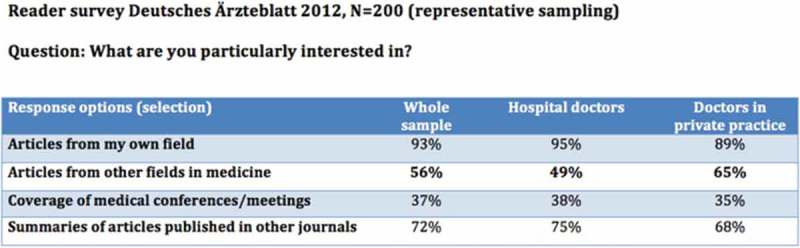
Results of the evaluation of CME articles in the journal of the German Medical Association (with permission from Professor Christopher Baethge).

Christopher Baethge MD, Chief Scientific Editor of Deutsches Ärzteblatt, the German Medical Association’s weekly journal and its bilingual international version Deutsches Ärzteblatt International presented compelling data from anecdotal evidence, reader feedback and a representative reader survey that German physicians from a range of specialties were reading CME articles outside their area of practice. The results of the survey [1] showed that 56% of physician readers were interested in articles from other fields (see Figure 2).

Professor Baethge concluded that his readers genuinely wanted to access interdisciplinary information rather than just accumulate CME points.

### Refreshment or redundancy

Martina Siedler, Head of Educational Publishing at Springer Medizin Verlag GmbH described a research project involving an analysis of evaluation data from 27,000 physicians who had accessed 547 different CME modules published in 29 Springer journals. The total number of evaluations in the analysis was 245,000 and the questionnaire focused on:

- the difficulty level of the CME modules,
- the reason for accessing the modules,
- the degree to which the CME module helped the participants to reinforce current knowledge, and
- the degree to which the CME module helped the participants to expand specialist knowledge.

**Figure 3.**
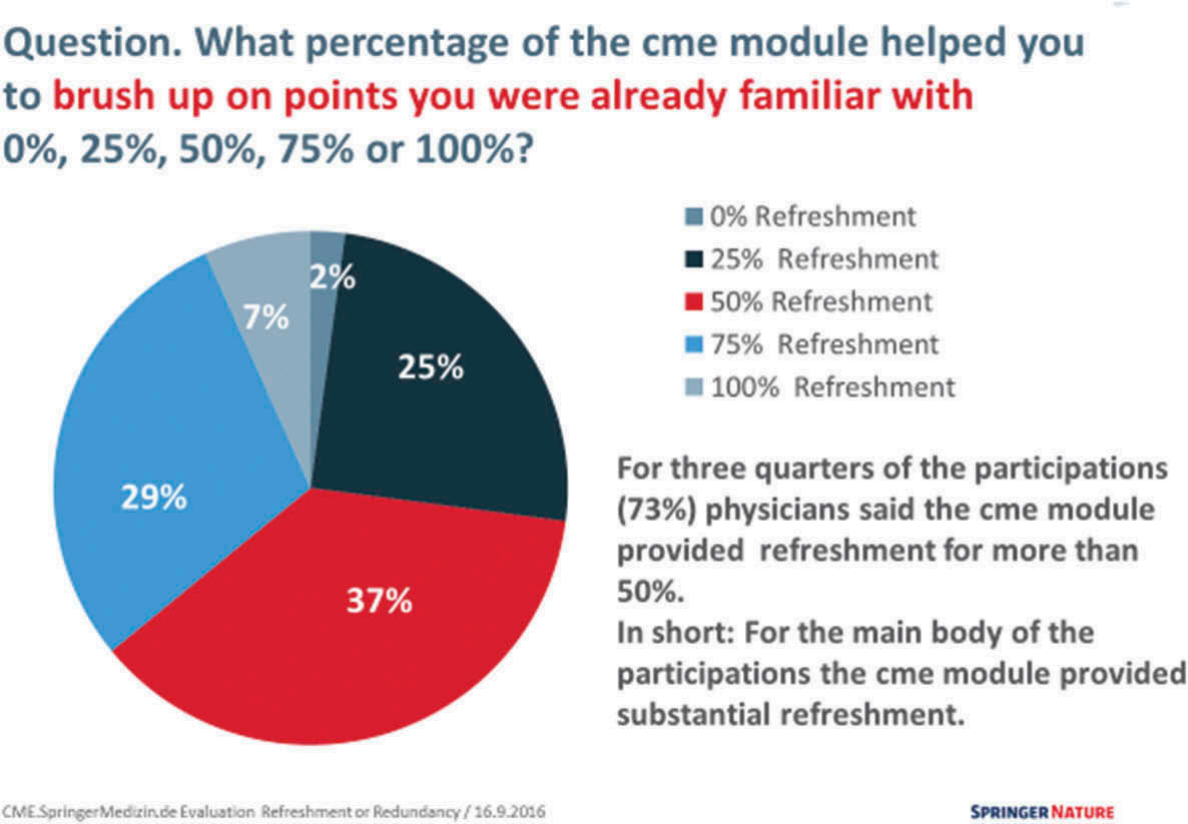
Refreshment of current knowledge via CME modules (with permission from Martina Siedler).

The results indicated that 91% of participants felt that the difficulty level was appropriate and that 75% accessed the content because of its relevance to their practice. For the majority of the participants (73%) the CME modules provided substantial refreshment (see Figure 3) and at least some new specialist knowledge (97%). Similar results appeared when the analysis drilled down to specific specialties, and, as might be expected, both the refreshment of current knowledge and expansion of specialist knowledge was slightly less among those participants with higher level qualifications.

### A refreshing attitude

Dr Bernd Hagen of Central Research Institute for Ambulatory Healthcare, Cologne, Germany presented details of a disease management programme involving targeted CME articles as part of a feedback system to improve prescription behaviour to conform with guidelines (a combination of ACE-inhibitors and beta blockers) among physicians in North Rhein treating patients with heart failure. The prescribing behaviour of those receiving the CME articles was compared with another cohort who did not receive the CME articles. The details of the study have previously been published in the *Journal of European CME* [2] and pointed to a long-term improvement of approximately 5% in adherence to guidelines by the participants receiving CME articles as part of their feedback. However, as pointed out by Dr Hagen and other conference participants during discussion, the effect of such interventions is small, and multiple CME interventions plus feedback may be needed to translate into significant improvements in patient care.

### Potential for change

Professor Reinhard Griebenow presented the data from an extensive evaluation project on behalf of Dr Peter Lösche, CEO, Academy for Training and Education, North Rhine Chamber of Physicians who was unable to attend the conference. More than 34,000 evaluation forms were analysed from participants from a range of specialties at live events occurring in North Rhineland prior to CME becoming mandatory.

The salient results from this self-assessment study were:

- that the relevance of the event topic to daily medical practice was the main impetus for attending an event,
- relevance of the event topic also determined how often participants had unanswered questions or were seeking some clear clinical strategy,
- these participants also demonstrated most self-perceived change,
- relevance of the event topic also affected how often participants did not benefit from the event,
- medical specialists participating in live educational events are generally open to change, and
- overall about 25% of participants experienced some sort of change in their strategy for clinical decision-making.

**Figure 4.**
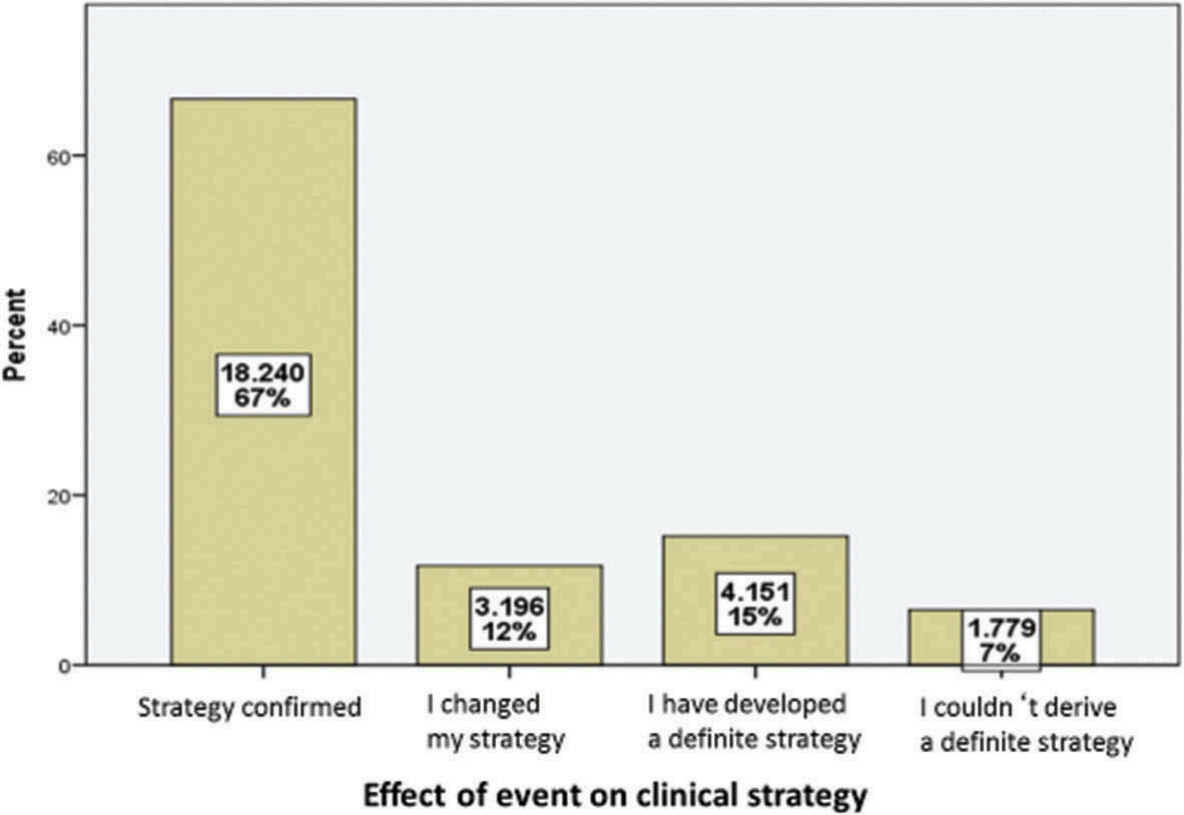
Changes in clinical strategy (with permission from Professor Reinhard Griebenow).

Although not quantifiable or studied in this analysis, discussion led to a consensus view that informal knowledge gained from dialogue with colleagues may be just as important as that gained from formal live events. Some effects measured are shown in Figure 4.

## Assessment of interprofessional teams

Dr Graham McMahon, President and CEO of ACCME joined the conference via Skype from Melbourne, Australia with a presentation on Interprofessional Teams in Healthcare. This was based on discussion of the value of teams, team characteristics and the assessment of teams with a real-world example provided of an Integrated Teaching Unit at a community teaching hospital in Boston, MA, USA.

Some of the benefits accruing from the formation of teams in healthcare were that they can:

- accomplish otherwise impossible goals,
- provide more solutions,
- identify flaws in solutions,
- develop a workplace community,
- add complementary skills, and
- provide growth in skills for individual team members.

**Figure 5.**
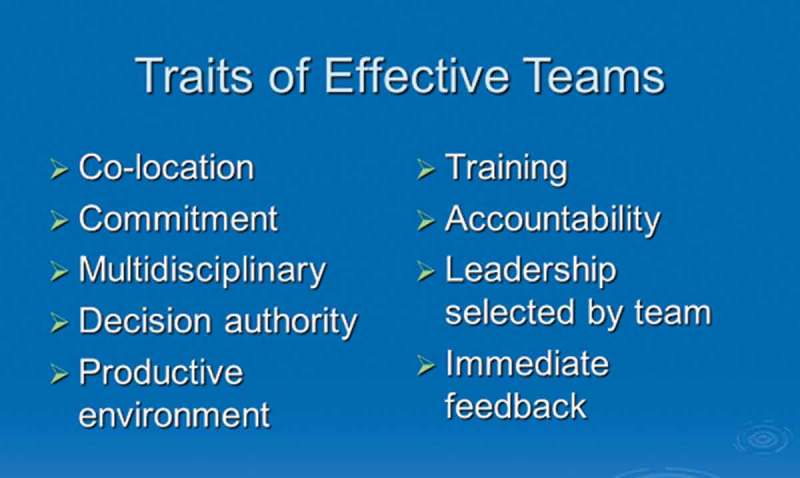
Traits of effective teams (reproduced with permission from Dr Graham McMahon).

Other important features needed to facilitate and maintain team performance ranged from the sharing of knowledge structures and mutual respect to effective communication and wise use of resources. Some traits of effective teams are shown in Figure 5.

In terms of the assessment of team performance, Dr McMahon suggested that surveys, observations, and outcomes measurements can be implemented and that, importantly, ongoing feedback improves performance.

## Assessment of providers

Day 1 concluded with a presentation by Kate Regnier, Executive Vice-President of ACCME who described its role as the body which accredits institutions i.e. providers that offer CME in the US and internationally. This contrasts with other accrediting bodies in USA and Europe where activities rather than providers are accredited. Part of ACCME’s mission is to promote standards for quality CME for physicians ultimately to improve medical care for patients and their communities. To meet this aspect of its mission, ACCME has implemented a peer review system of assessment to ensure that accredited providers adhere to the standards mentioned above.

**Figure 6.**
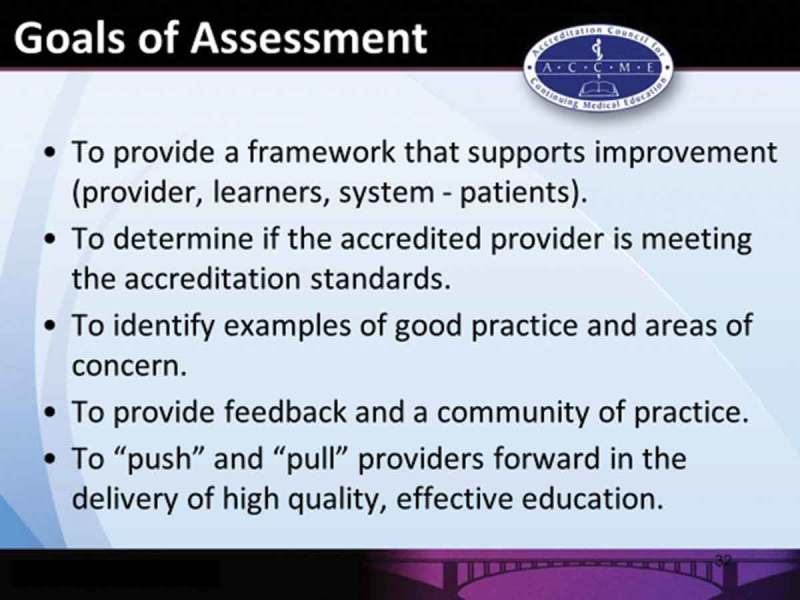
ACCME’s goals of provider assessment (reproduced with permission from Kate Regnier).

The framework for this system is a set of criteria and a monitoring programme that allows providers to support quality patient care via the continuum of medical education. This is achieved through data collection and reporting by providers and support provided by ACCME through education and the provision of resources. The sources of data used by ACCME to make accreditation decisions are a self-study report, evidence of performance-in-practice (activity files) and an accreditation interview. Decisions on accreditation may range from non-accreditation to provisional accreditation (two years), accreditation (four years) and accreditation with commendation (six years). The overall goals of this assessment system for CME providers is shown in Figure 6.

An interesting topic of discussion arose on the differences between the accreditation process in Europe and that supported by ACCME, particularly because the first non-US provider accredited by ACCME was present. The main point emphasised was that educational content is separated from any aspect of pharmaceutical or device industry promotion.

## Day 2

The main headings for the topics covered during Day 2 of the conference were as follows:

- assessment of knowledge,
- assessment of practical skills,
- evaluation of CME/CPD,
- evaluation of mega events, and
- assessment and licensure.

## Assessment of knowledge

### MCQ writing

The opening session of Day 2 began with a detailed description of steps involved in the production of appropriately structured multiple choice questions (MCQs) for the European Society of Cardiology by Dr Renée van den Brink of the Academic Medical Centre in Amsterdam. These MCQs are designed to test knowledge of topics in the core curriculum for general cardiologists, but not directly observed procedural skills. The technical aspects of creating such MCQs were explained with the main components being:

- a clinically-based stem,
- the question itself,
- five options for the candidate to choose from, one of which is the “best” answer and the others of which are plausible alternatives (distracters), and
- inclusion of a still image or video clip if appropriate.

The question should be direct, unambiguous and address a single issue.

In addition, the avoidance of negatively worded questions e.g. “what is least likely” and avoidance of “keying” to the correct answer was emphasised. Details of the question submission to a question bank and review process were also described which highlighted the fact that writing a good, clinically relevant MCQ for the European Exam for General Cardiology (EEGC) is difficult, time-consuming and requires highly accurate peer review by several cardiologists from different countries.

### European examination general cardiology

**Figure 7.**
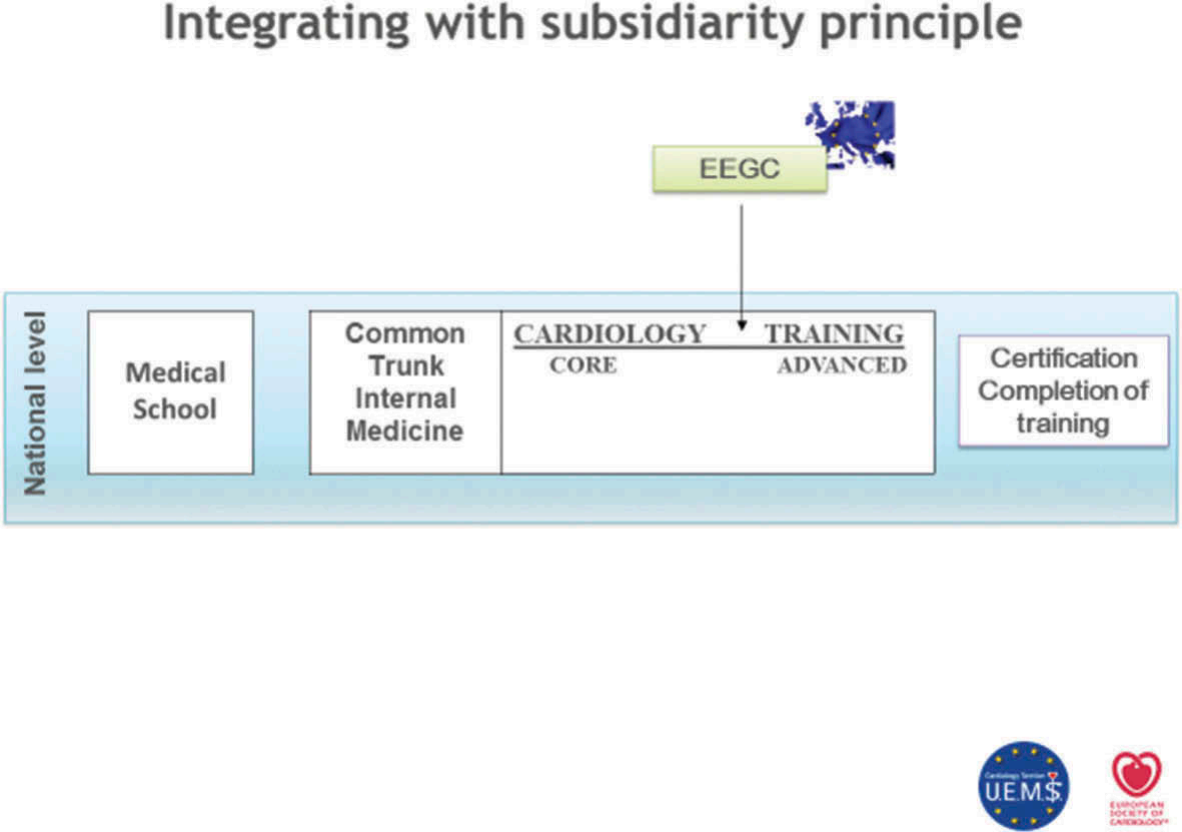
Where the EEGC fits within the training of specialist cardiologists in Europe (with permission from Dr Jim Hall).

Dr Jim Hall UEMS-Cardiology Section VP Training provided further detail of the use of the MCQs in the examination as an assessment strategy for specialist trainees as part of the training requirements in Europe. This is a knowledge-based assessment that fits within the spectrum of European cardiology training as shown in Figure 7.

This is a pass/fail examination comprising 120 MCQs during a three-hour exam taken at an independent testing centre. Candidates are urged to prepare for the exam by accessing as wide a range of resources as possible from textbooks and journals to online educational materials. The exam is conducted by national cardiology societies in collaboration with the European Cardiology Society and the Cardiology Section of UEMS. Overall governance of the procedures is conducted by various linked groups namely the Question Writing Group, the Question Selection Group, the Standard Setting Group and Exam Performance Review Group. The exam has expanded from a pilot in the UK in 2009 to seven countries in 2016 and plans for a rollout to five more countries within the next two years.

### CME in print media

Professor Reinhard Griebenow described a study of CME in print media in Germany which represents about 1% of the 360,000 activities which require 10 MCQs per unit of education. An analysis of responses to MCQs associated with articles in the *Journal of the German Medical Association* and a series of specialist journals was conducted. The data indicated that a pass rate of 99% was achieved for the single journal articles and 97.5% for the review type articles in the specialist journals. Discussion focused on the fact that mismatches existed between the information provided and the MCQs used as the assessment and led the presenter to conclude that, notwithstanding the quality of the MCQs, such knowledge-based assessment of CME in print (and digital) media is of limited value as a test and is more a measure of participation.

## Assessment of practical skills

### Angiography simulation

Professor Wolfram Voelker of the Department of Internal Medicine, University of Wuerzburg described the current needs of trainees in interventional cardiology dealing with challenges such as patients with multiple co-morbidities undergoing complex procedures using complicated technical equipment in team-based situations under difficult economic conditions.

This has led to a need for training to match the advances in the equipment and approaches to teamwork. A comparison with the aviation industry, where simulation has long been a feature of skills mastery training, with the time-consuming apprenticeship model for interventional cardiologists, has led to consideration of virtual reality simulation as part of their training. This approach is designed to help trainees learn the proper sequence of catheterisation steps, improve hand-eye-coordination, become more prepared to deal with complications and develop enhanced visual and spatial orientation.

**Figure 8.**
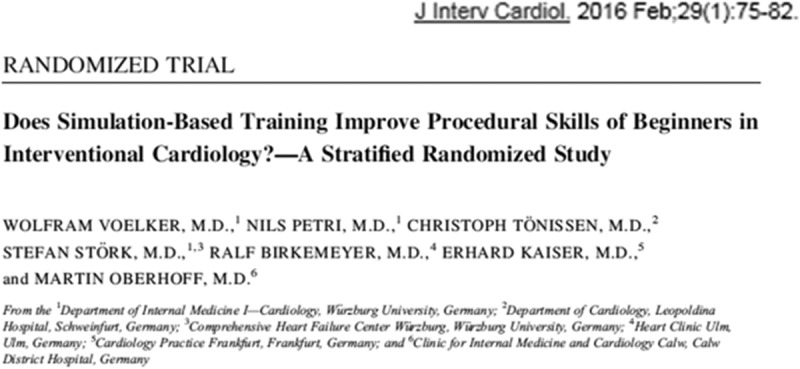
Study on simulation-based training.

Studies (see Figure 8) are ongoing to assess whether mentored simulation-based catheter training can improve the performance level of novices in interventional coronary procedures. A report from the British Cardiovascular Society has recommended this approach and that such training should be recognised as a component of CME requirements in Europe.

Although early results in both group and individual assessment from this approach seem promising, Professor Voelker stressed the need for more studies to help improve the validity of using simulation-based learning in this field.

### Echocardiography

**Figure 9.**
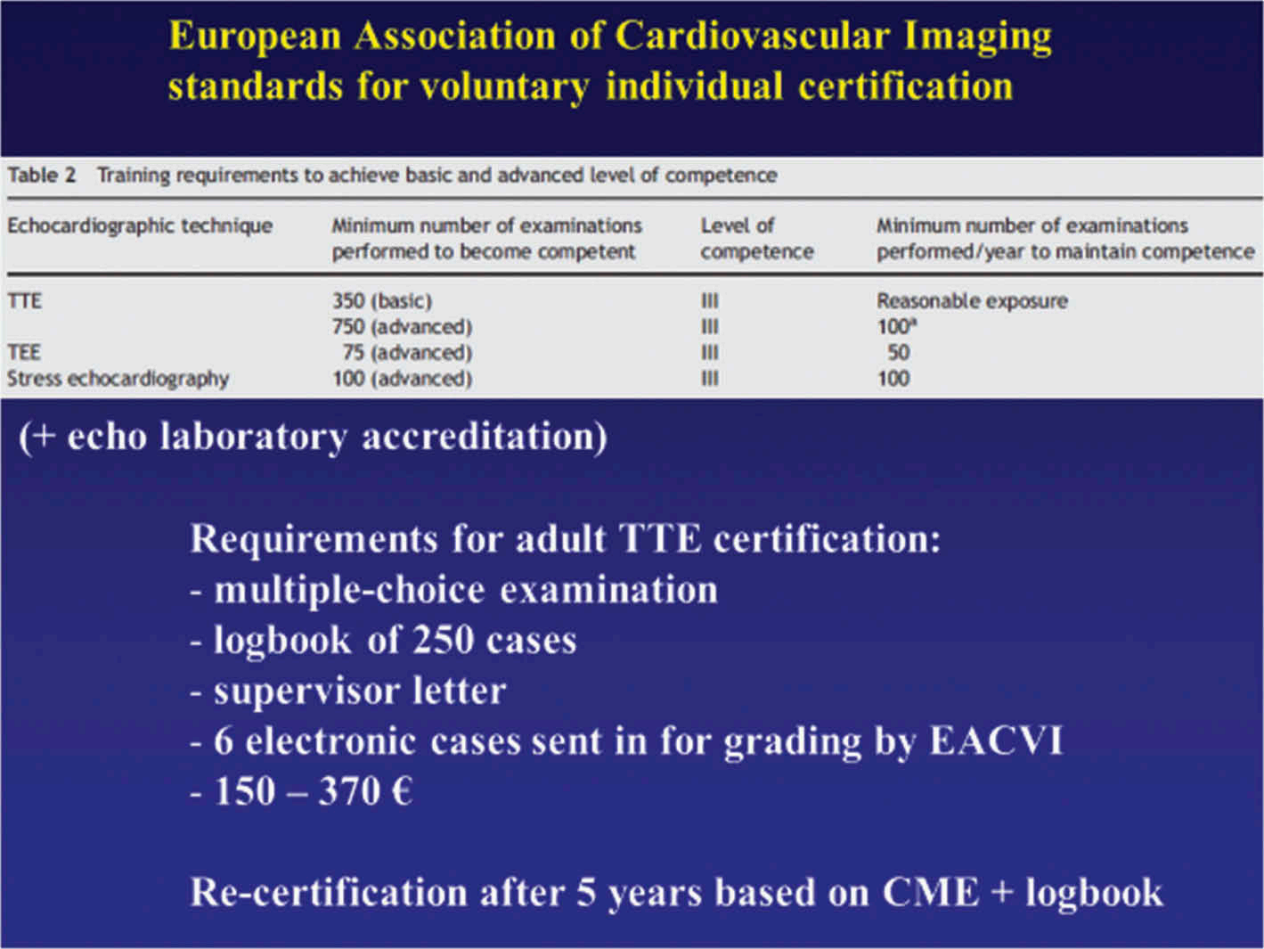
EACVI standards for echocardiography certification (with permission from Professor Frank Flachskampf).

Professor Frank Flachskampf from Uppsala, Sweden described the procedures used in evaluation and quality control in cardiology fellowship training in echocardiography in both Sweden and Germany. The practical skills involved include the technical aspects of operating the machines efficiently to obtain good images as well as the communication skills to interact with patients. He illustrated similarities and differences between Sweden and Germany in the approach to training. In Germany training may occur in hospital settings as well as in private practice, whereas Sweden’s system relies mainly on the National Health Service. The rationale for setting up echocardiology training in Sweden has not been subject to any specific regulation, as is the case in Germany. From a European perspective, a pan-European organisation does exist – the European Association of Cardiovascular Imaging (EACVI), which is a subgroup of the European Society of Cardiology and has produced a set of standards for voluntary individual certification as shown in Figure 9.

The numbers applying for this certification are increasing but still represent a small fraction of European cardiologists. Simulation training was also discussed as a possible option for the future and Professor Flachskampf pointed out that, despite differences in the level of scrutiny and regulation between Germany and Sweden, there was no significant difference in the quality of images being produced, perhaps due to the influence of peer review of diagnostic procedures being used as a component of assessment.

### Cardiac magnetic resonance imaging

The final example of practical skills assessment was presented by Dr Mark Westwood, Director of Education at Bart’s Heart Centre in London, UK who discussed the three-level CMR certification provided by EACVI, the goal of which is to develop a standard of training in CMR that is mutually accepted by national and European authorities and recognised internationally. Although not compulsory, but an adjunct to national requirements for reporting and signing CMR studies, the certification is designed to bring some measure of professional recognition and allow trainees to test their knowledge and skills against internationally set standards.

**Figure 10.**
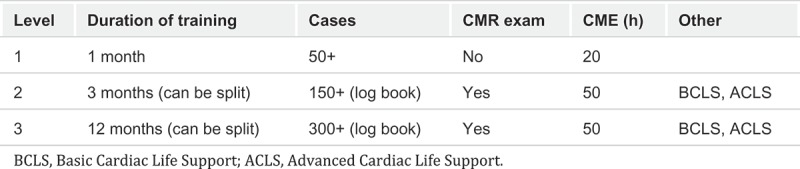
Summary of recommendations for individual certification in CMR (adapted with permission from Dr Mark Westwood).

Certification is available at three competency levels, ranging from core CMR training at level 1 to levels 2 (supervised) and 3 (autonomous), both of which require a pass in the European CMR exam and onsite training with the completion of a logbook containing original, anonymised CMR studies. These must be accrued within a 24-month period before and after passing the written exam. Dr Westwood noted that some training centres may not have the capacity for trainees to accrue the required number of cases so dispensation is allowed for candidates to receive training in other centres and to use cases from these centres with a limit of 100 cases for level 2 and 200 for level 3 certification. The recommendations for the certifications are outlined in Figure 10.

## Evaluation of CME/CPD

### A provider’s view

**Figure 11.**
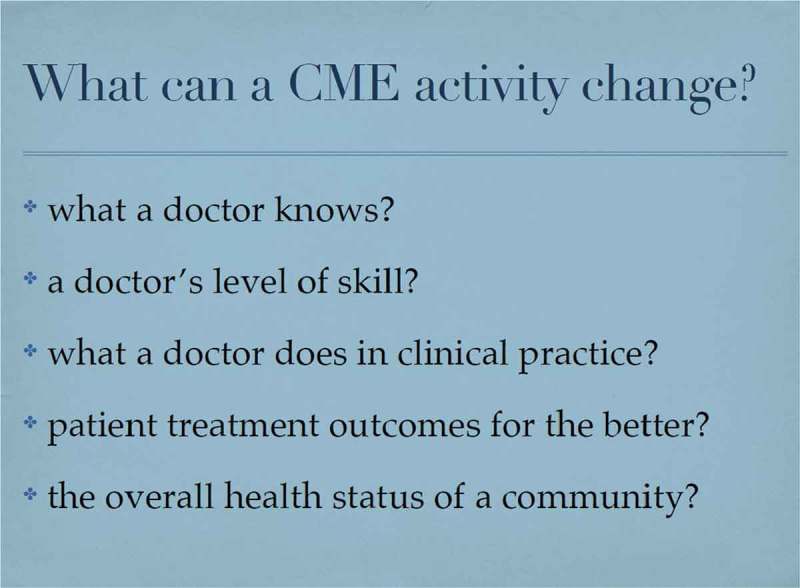
What is feasible for providers to evaluate? (with permission from Eugene Pozniak).

Eugene Pozniak, Managing Director of Siyemi Learning, the first ACCME accredited CME provider outside USA, posed the general question “What is feasible in evaluating CME/CPD?” then expanded this to consider the questions shown in Figure 11.

**Figure 12.**
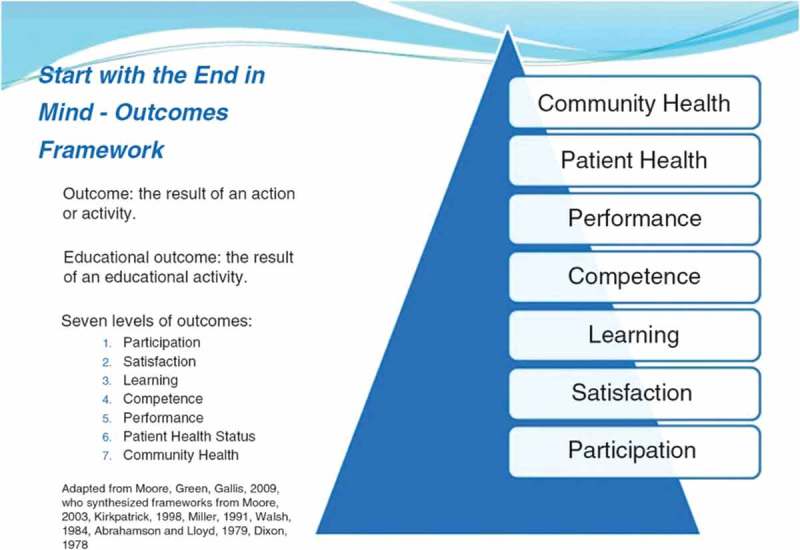
CME outcomes measurements (adapted with permission from Eugene Pozniak).

He began with the thesis that, in CME/CPD, knowledge alone is not enough to effect change (e.g. smoking cessation) but that outcome measurements can be conducted at several levels as illustrated in Figure 12.

Eugene Pozniak put forward the notion that it may sometimes be better to use change in behaviour, rather than knowledge, as the starting point and that at least level 4 (competence) as an outcome should be a goal in the planning and implementation of many CME activities. This approach was summarised in his exhortation to start the education planning process with the outcome being sought and plan backwards from that point as outlined by Moore et al. [3].

## Evaluation of mega events

### American College of Cardiology

Ellen Cohen, Director, Accreditation, Certification & MOC for ACC in Washington, DC, USA presented details of the evaluation procedures for the ACC’s annual meeting which attracted over 18,000 attendees at their 2016 conference. The conference utilised 20 different session formats addressing 12 different clinical learning pathways over a two-day period. The assessment plan was based on measuring attendee experiences and learning from an overall (required) and session level (optional) perspective, which was administered through an online portal and a smartphone application.

A follow-up survey was also conducted to measure performance by means of a two-month outcomes survey to evaluate long-term learning improvement with a view to understanding long-term primary practice change and potential barriers to implementation. Whilst data from this follow-up survey would be very useful, the response rate was very small (5.4%) and represents a challenge for the organisation to motivate attendees to complete an outcomes survey.

**Figure 13.**
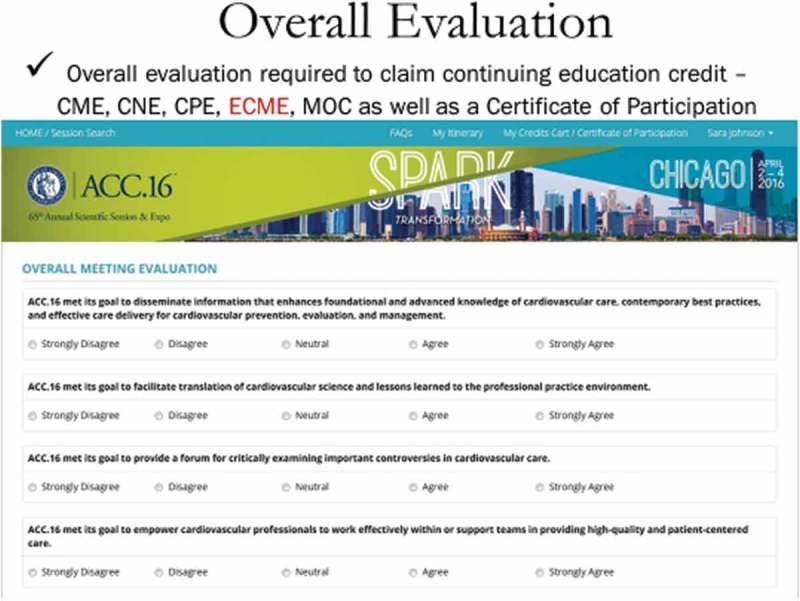
ACC overall evaluation template (with permission from Ellen Cohen).

The Annual Scientific Session (ACC.16) is evaluated upon a set of overall programme goals as illustrated in Figure 13. Evaluation is also based upon the Accreditation Council for Continuing Graduate Education (ACGME) core competencies, which include: patient care, medical knowledge, practice-based learning and improvement, interpersonal and communication skills, professionalism and systems-based practice. The evaluation results indicated that medical knowledge scored the highest and interpersonal and communication scored the lowest in the evaluation of ACC.16.

Discussion among participants confirmed that a certain degree of evaluation and assessment occurs during some of the sessions using pre-and post-testing as well as an audience response system.

### German Cardiac Society

Konstantinos Papoutsis, CEO of the German Cardiac Society reiterated many of the points of previous presentations in their approach to the evaluation of their annual and autumn congresses where they were considering the quality of organisational aspects, relevance of content and evaluation of presenters.

The formal (paper-based) evaluation of their larger meetings – 9000 participants for the annual meeting and 2700 for the autumn meeting – was deemed to be cumbersome and of little value compared with the procedures and results associated with the smaller Academy meetings organised by the society. In this case a higher response rate was achieved with smaller groups up to a maximum of 60 attendees who experienced a closer engagement with faculty and colleagues and had further motivation through having to pay a fee to attend unlike the annual and autumn meetings which are free to attend for members.

Because of these experiences the society has decided to abandon the formal evaluation of the larger meetings and rely on the informal feedback from attendees at these meetings. Meanwhile the enhanced practicability will allow smaller meetings to continue to be evaluated with plans to look at streamlining the evaluation procedures.

### German Academy for Ophthalmology

Julia Hörster of the Association of German Ophthalmologists (BVA) in Düsseldorf completed the sessions on mega event evaluation by describing their system for a five-day annual meeting with almost 6000 attendees representing physicians, nurses, patients and students. Participants book spaces at sessions on an individual basis via an online platform and receive a name tag with a QR-Code which is scanned for entry to booked sessions, where they are presented with evaluation sheets. Those claiming CME credits have these awarded as a result of the scanning system.

**Figure 14.**
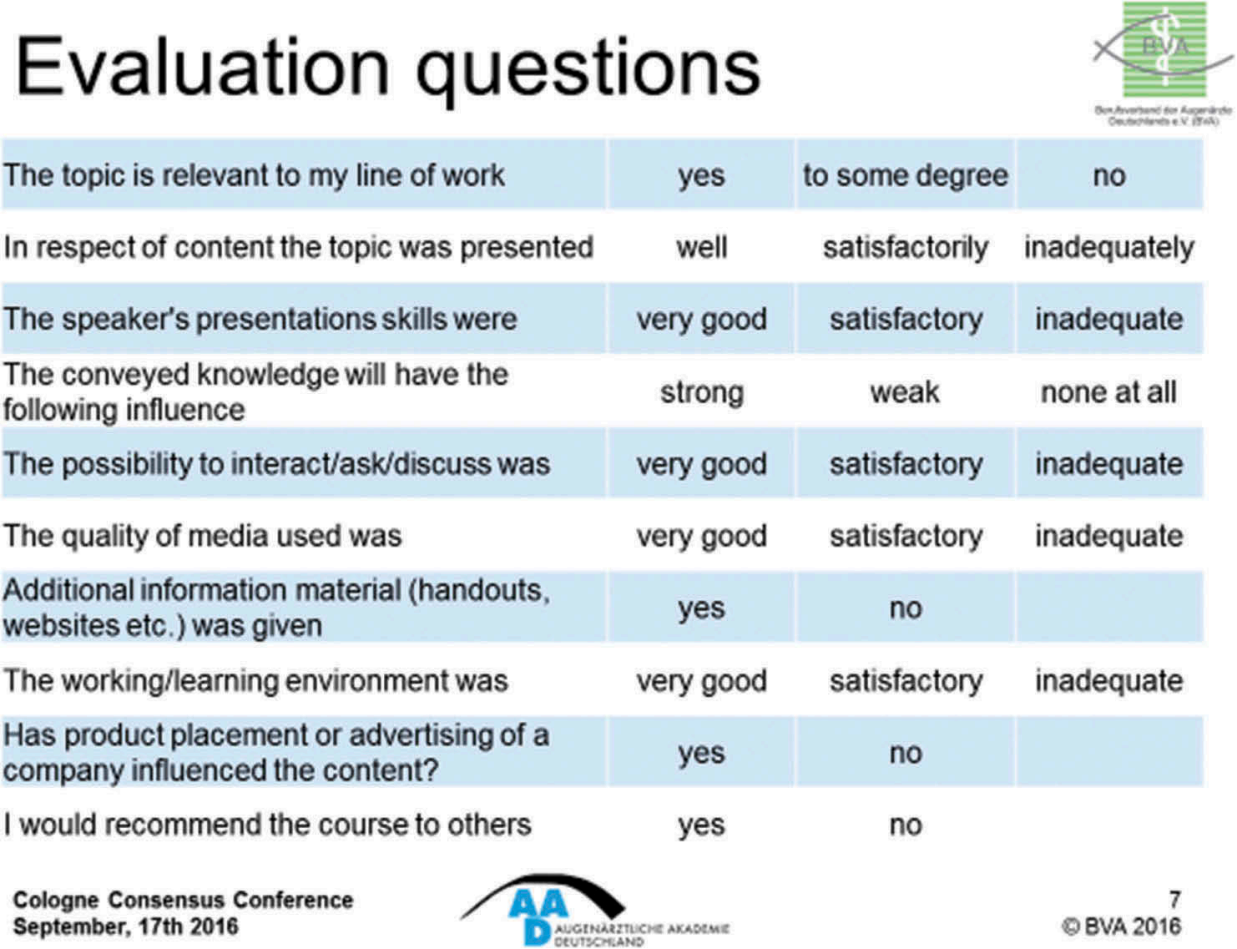
AAD evaluation questions (with permission from Julia Hörster).

The evaluation questions with a three-point scale (limited to 10) as shown in Figure 14 have been produced by a committee on the basis of recommendations derived from a cross-sectoral working group on CME evaluation in the early 2000s.

BVA consider evaluation as an essential tool to assess conference content and provide an objective tool to suggest improvements and amendments to courses, organisation and conduct. Future plans include the introduction of a TED-type voting system via smartphone in 2017 in an attempt to increase participation rates from the current figure of approximately 53%.

## Assessment and licensure

### MOC programme: American Board of Internal Medicine (ABIM)

Marcie Bonilla, Director of Programme Operations at the American Board of Internal Medicine outlined how ABIM fits under the umbrella organisation of the American Board of Medical Specialties with its own range of specialities being designated as coming under the heading of Internal Medicine. The main thrust of the ABIM’s MOC programme is to allow physicians to demonstrate to the public and their peers their currency with medical knowledge and practice throughout their careers. The requirements for MOC can be summarised as follows:

- the completion of approved external activities, including CME activities produced by accredited providers,
- the completion of ABIM-produced activities,
- preparation for and taking the ABIM secure exam,
- successful completion of accredited fellowship training, and
- passing a secure exam every 10 years.

The ABIM is currently exploring more frequent, less burdensome assessment options and as part of this process has collaborated with ACCME with the following benefits accruing:

- ABIM’s medical knowledge recognition programme can be incorporated into ACCME’s Provider and Activity Reporting System (PARS),
- the collaboration between ABIM and ACCME came about in response to the needs of physicians and CME providers to be able to give credit for activities that physicians were already doing, and
- CME providers can register CME and MOC activities at the same time, in a single system.

**Figure 15.**
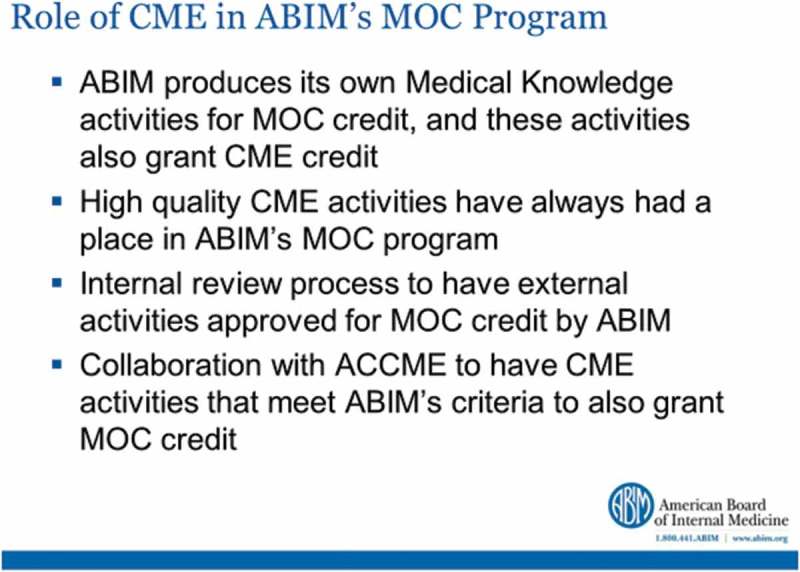
CME and the ABIM MOC programme (with permission from Marcie Bonilla).

This collaboration has expanded the options available to physicians and allowed CME providers to offer lifelong learning activities for MOC in a streamlined process. The link between CME and MOC is regarded as highly important by ABIM and summarised in Figure 15.

### MOC programme: Royal College of Physicians and Surgeons of Canada

Mya Warken, Senior CPD Accreditation Specialist at Royal College of Physicians and Surgeons of Canada discussed the relationship between physician licensure and CPD in Canada and the relationship between the maintenance of certification (MOC) programme’s assessment requirements and licensure in Canada. She described the staggered implementation of mandatory CME in Canada starting in 2007 with the provinces of Quebec and Saskatchewan, to the current situation where most of the provinces and territories require physicians to participate in CPD for licensure.

The regulation of medical practice in Canada is the responsibility of 13 provincial and territorial medical regulatory authorities (MRAs). The MRAs are members of the Federation of Medical Regulatory Authorities of Canada (FMRAC): a national organisation which represents the MRAs by promoting collaboration and standardisation. There are several MRA-approved CPD programmes including:

1. the Royal College’s Maintenance of Certification programme, requiring 400 credits during each five-year cycle,
2. the College of Family Physicians of Canada’s Mainpro+ programme requiring 250 credits over a five-year cycle, and
3. some provincial CPD programmes, e.g. Ontario and Quebec.

The Royal College’s MOC programme has been evolving since 1994 and currently consists of three sections: group learning, self-learning and assessment with various numbers of credits available for CPD activities recognised in each section. Participation in activities for each section must be documented in an e-portfolio. In 2015 43% of MOC programme participants recorded credits in the “Assessment” section, the lowest participation rate among the three sections, despite having the highest credit value at three credits per hour. For activities to be included in Section 3, learners must receive data and feedback for example by collecting data, responding to assessment questions, or participating in simulated scenarios. They must also review data, analyse results and participate in debriefing or reflection sessions to develop learning plans to address identified needs.

The Royal College is encouraging CPD providers to explore ways to increase participation in Assessment activities such as including knowledge assessment within group learning, offering simulation courses during annual meetings and utilising chart audit tools.

**Figure 16.**
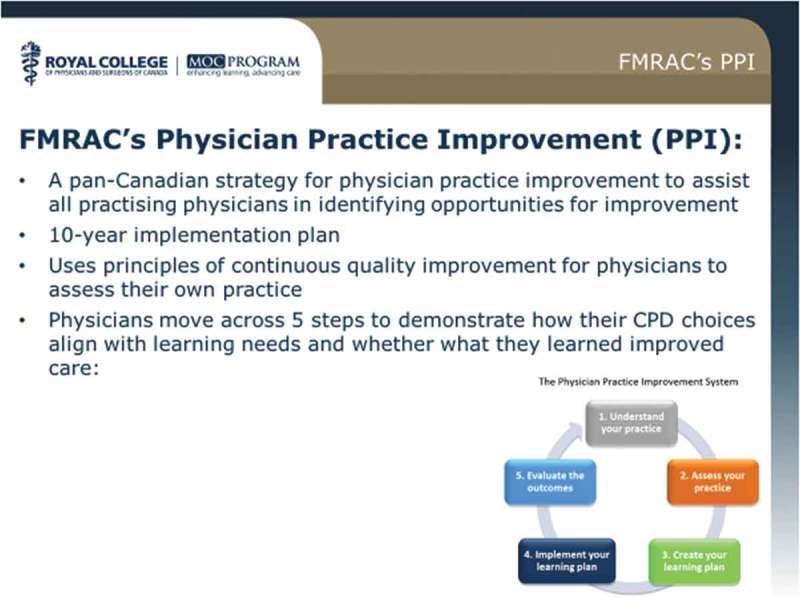
FMRAC’s physician improvement plan.

Future considerations for the MOC programme are based on a transition to a competency-based model for CPD with less emphasis on participation in learning activities simply for credit and more on measurable outcomes in practice improvement. Figure 16 illustrates part of this strategy for physician practice improvement.

(http://fmrac.ca/wp-content/uploads/2016/04/PPI-System_ENG.pdf)

## Take-home messages

The wide range of approaches to the topics covered in the 2016 Cologne Consensus Conference provided participants with a number of useful messages, such as:

- start at the endpoint when planning CME activities;
- the individual should be the target of all our work;
- a precise test is an illusion;
- move away from MCQs;
- trust our colleagues’ professionalism;
- accredit informal as well as formal learning; and
- the team approach is important.

The diversity of professional expertise among those present and the wide-ranging discussion among North American and European colleagues made this a stimulating and rewarding experience for all concerned. The 2017 conference will focus on the topic of Interprofessional CME/CPD under the auspices of ECSF, the Royal College of Physicians and Surgeons of Canada and ACCME. The complete set of presentations from the 2016 conference may be viewed on the ECSF website (www.e-cs-f.org).
